# Prune belly syndrome in surviving males can be caused by Hemizygous missense mutations in the X-linked Filamin A gene

**DOI:** 10.1186/s12881-020-0973-x

**Published:** 2020-02-21

**Authors:** Nida S. Iqbal, Thomas A. Jascur, Steven M. Harrison, Angelena B. Edwards, Luke T. Smith, Erin S. Choi, Michelle K. Arevalo, Catherine Chen, Shaohua Zhang, Adam J. Kern, Angela E. Scheuerle, Emma J. Sanchez, Chao Xing, Linda A. Baker

**Affiliations:** 1 Department of Urology, University of Texas Southwestern Medical Center, 5323 Harry Hines Blvd, Dallas, TX 75390 USA; 2 Broad Institute of MIT and Harvard, Cambridge, MA USA; 3 Department of Pediatrics, University of Texas Southwestern Medical Center, 5323 Harry Hines Blvd, Dallas, TX 75390 USA; 4 McDermott Center for Human Growth and Development, Department of Bioinformatics, Department of Clinical Sciences, University of Texas Southwestern Medical Center, 5323 Harry Hines Blvd, Dallas, TX 75390 USA; 5 Children’s Health Dallas, 2350 N. Stemmons Freeway, Suite F4300, Dallas, TX 75207 USA

**Keywords:** Prune belly syndrome, FLNA, Sequencing

## Abstract

**Background:**

Prune belly syndrome (PBS) is a rare, multi-system congenital myopathy primarily affecting males that is poorly described genetically. Phenotypically, its morbidity spans from mild to lethal, however, all isolated PBS cases manifest three cardinal pathological features: 1) wrinkled flaccid ventral abdominal wall with skeletal muscle deficiency, 2) urinary tract dilation with poorly contractile smooth muscle, and 3) intra-abdominal undescended testes. Despite evidence for a genetic basis, previously reported PBS autosomal candidate genes only account for one consanguineous family and single cases.

**Methods:**

We performed whole exome sequencing (WES) of two maternal adult half-brothers with syndromic PBS (PBS + Otopalatodigital spectrum disorder [OPDSD]) and two unrelated sporadic individuals with isolated PBS and further functionally validated the identified mutations.

**Results:**

We identified three unreported hemizygous missense point mutations in the X-chromosome gene *Filamin A* (*FLNA*) (c.4952 C > T (p.A1448V), c.6727C > T (p.C2160R), c.5966 G > A (p.G2236E)) in two related cases and two unrelated sporadic individuals. Two of the three PBS mutations map to the highly regulatory, stretch-sensing Ig19–21 region of FLNA and enhance binding to intracellular tails of the transmembrane receptor β-integrin 1 (ITGβ1).

**Conclusions:**

FLNA is a regulatory actin-crosslinking protein that functions in smooth muscle cells as a mechanosensing molecular scaffold, transmitting force signals from the actin-myosin motor units and cytoskeleton via binding partners to the extracellular matrix. This is the first evidence for an X-linked cause of PBS in multiple unrelated individuals and expands the phenotypic spectrum associated with *FLNA* in males surviving even into adulthood.

## Background

Prune Belly Syndrome (PBS), also known as Eagle-Barrett or Triad Syndrome (MIM#100100) is a congenital myopathy with highly variable multisystem phenotypic severity, affecting 1 in 25,000 individuals [1–3]. The classic triad defining PBS, which almost exclusively affects males, includes 1) the wrinkled, prune-like ventral abdominal skin with underlying flaccid hypoplastic skeletal muscle, 2) urinary tract dilation including megacystis and megaureter with poorly contractile smooth muscle, and 3) bilateral intraabdominal cryptorchidism. However, the PBS phenotype has been categorized into isolated PBS, syndromic PBS, and PBS-plus, based on the absence or presence of genetically defined syndromes or additional malformations outside the classic triad, respectively [2]. Megacystis +/− oligohydramnios is prenatally diagnosed by ultrasound in most cases of PBS with premature birth in 43% and neonatal death in 23–45% [1, 4]. Survivors typically have normal cognition but battle multi-organ dysfunction with ~ 50% developing chronic renal insufficiency or end-stage renal disease [4–7]. It has been estimated that there are 1494 males under the age of 18 years living with PBS in the US [8].

Historically, the cause of PBS was attributed to a mechanical in utero *bladder outlet obstruction*. However, a true urethral obstruction is observed in only 10–20% of PBS autopsy cases and does not account for situations where significant abdominal muscular hypoplasia is observed with only a mild urogenital defect [9]. Thus, an alternative etiological hypothesis is that PBS is a consequence of a *mesodermal field defect* of unknown cause [10]. Most PBS cases are sporadic and no environmental factors have been implicated as causal. Rare PBS case reports have noted abnormal karyotypes, including ring X chromosome, trisomy 13, 18 and 21 [11–18]. There are three pieces of evidence that suggest a genetic basis influenced by X-linked recessive or sex-limited autosomal recessive genetic factor(s) [19]. First, rare concordant PBS twin gestations have been reported [20–22]. Second, > 95% of cases are male. Lastly, there have been 12 published multiplex pedigrees without causal genes identified in most [19–21, 23–31]. More recently, five autosomal genes, including *CHRM3, HNF1β, ACTA2, ACTG2* and *STIM1*, have been reported with potentially causal DNA variants, including structural, copy number, and single nucleotide variants, however these genes each only account for one or two PBS cases or one PBS multiplex consanguineous kindred [32–38]. Moreover, none of the currently suggested candidate genes fit an X-linked recessive mode of inheritance and functional data is lacking for many of these candidate genetic variants.

In this report, we identify four PBS-affected individuals, two sporadic and two from a multiplex kindred, to have mutations in the X-chromosome gene, *FLNA*. *FLNA* encodes for a large actin-binding scaffolding protein that functions in diverse cellular processes including cell migration, wound healing and organ development [39, 40]. Mutations in *FLNA* underlie a wide spectrum of human disease phenotypes broadly termed the filaminopathies that include periventricular nodular heterotopia (PVNH), the otopalatodigital spectrum disorders (OPDSD) which includes Melnick-Needles Syndrome (MNS) and frontometaphyseal dysplasia (FMD) as well as X-linked cardiac valvular dystrophy (XCVD), among others. Given the X-linkage, phenotypic severity is highly variable in females depending on the exact *FLNA* variant and the impact of skewed X inactivation. In contrast, most male *FLNA* mutation carriers, especially those with OPD2 and MNS, die in utero or early antenatally [41, 42]. We now add a new cohort of males surviving even into adulthood with *FLNA* missense mutations, expanding the spectrum of *FLNA* phenotypes to include males with syndromic PBS with OPDSD or those only with isolated PBS.

## Methods

### Study subjects

Internationally, we prospectively enrolled individuals with PBS and their family members in our IRB-approved Pediatric Genitourinary DNA Repository starting in 2001. Informed consent was obtained from all individuals included in the study which was approved by the institutional review board at UT Southwestern Medical Center and all procedures followed were in accordance with the ethical standards of the relevant committees on human experimentation. Patients’ medical records were retrospectively reviewed and in person and/or telephone interviews were performed to obtain medical, surgical and family history. Medical photographs were made and reviewed by a clinical geneticist (AES). Each individual with PBS was assigned a PBS severity score using the RUBACE (R: renal, U: ureter, B: bladder/outlet, A: abdominal wall, C: cryptorchidism, E: extra-genitourinary) phenotyping scoring system developed by our group to better grade disease severity and categorize patients into isolated PBS, syndromic PBS or PBS-Plus groups [2].

#### Whole exome sequencing

Lymphocyte genomic DNA was extracted according to standard procedures from participants using the Puregene DNA isolation kit (Gentra/Qiagen) or from saliva (Oragene). Paired-end Whole Exome Sequencing (WES) was performed at the UTSW McDermott Next Generation Sequencing Core using the Illumina HiSeq2500. Library preparation was done using the Illumina SureSelect DNA Sample prep kit and capture with the Illumina SureSelect Exome Enrichment kit. Data processing and analysis was performed by the UTSW McDermott Center Bioinformatics group. Adaptor removal and sample demultiplexing was done using CASAVA, BWA was used for alignment to the human genome (GRCh37/hg19), mapped reads were processed, sorted and underwent duplicate removal using Samtools and PICARD, and GATK was used for quality control, including realignment around insertions and deletions and base quality score recalibration. Variant calling was performed using training sets from data from the 1000 Genomes Project, Omni 2.5 M SNP microarray and HapMap phase 3.3. Variant call files were filtered to exclude those with a minor allele frequency (MAF) too high to account for PBS from public databases including, ExAC, gnomAD, 1000 genomes, (ExAC AF < 0.005 for homozygous variants and ExAC AF < 0.00005 for heterozygous or hemizygous variants) and only functional variants (predicted to alter mRNA splicing or amino acid sequences) were included for further analysis.

#### Gene expression studies

mRNA expression was assessed across normal adult human pooled RNA samples (BioChain) using qPCR following standard methods. Briefly, 1 μg of RNA was reverse transcribed (BioRad iScript cDNA synthesis kit) with oligo (dT) and random hexamer primers. Gene specific primers (*FLNA*: for 5′- CTGTCACAGTGTCAATCGGAGG and rev 5′- TCGAAAGTGCCGTCCTCATT; *ITGB1*: for 5′- CCTACTTCTGCACGATGTGATG and rev 5′ - CCTTTGCTACGGTTGGTTACATT) were used to amplify mRNA using KAPA SYBR green mix on the CFX Connect Real Time System (BioRad). Differential gene expression was calculated via the delta delta CT method and normalized to *GAPDH* and all samples were run in triplicate (*n* = 3). Significance was calculated by t-test using Graph Pad Prism 7.03 software.

Immunohistochemistry and histology: Bladder biopsy, obtained from consenting normal pediatric individuals at the time of bladder surgery to correct refluxing ureters (*n* = 4), was fixed in 10% neutral buffered formalin and routinely processed for paraffin embedding. Briefly, tissues were dehydrated in sequentially increasing ethanol concentrations ending with xylene and infiltrated with paraffin. Tissues were embedded in paraffin and sectioned at 4uM. Hematoxylin and eosin staining was used to assess tissue morphology. Immunohistochemistry was performed (FLNA #HPA01115 (Sigma) and ITGβ1 #9699 (Cell Science Technology)) at the UTSW Tissue Management Shared Resource. Staining was automated on the Dako Autostainer Link 48 system to ensure identical staining conditions. Slides were scanned using the Hammamatsu nanozoomer 2.0.

#### Binding assays

Full length human FLNA with C-terminal GFP in pcDNA3 (Calderwood Lab, Yale University) was used as template to introduce all identified FLNA mutations with the QuikChange II XL kit (Agilent) and verified by Sanger sequencing of the complete FLNA insert to rule out any additional mutations. For analysis of binding of full length FLNA to integrin, CHO (Chinese Hamster Ovary) cells were transiently transfected with FLNA plasmids using Lipofectamine 3000 (Invitrogen). Binding was assessed to purified integrin tails, β1 wild type, β1 Y788A, and β7 (Calderwood Lab, Yale University) following protocols previously described [43] with the modifications that 10 mM imidazole was included in buffer XT and the beads were washed three times.

## Results

### Clinical findings of PBS patients (Figs. 1 and 2, supplemental figs. 1 and 2)

**Fig. 1:**
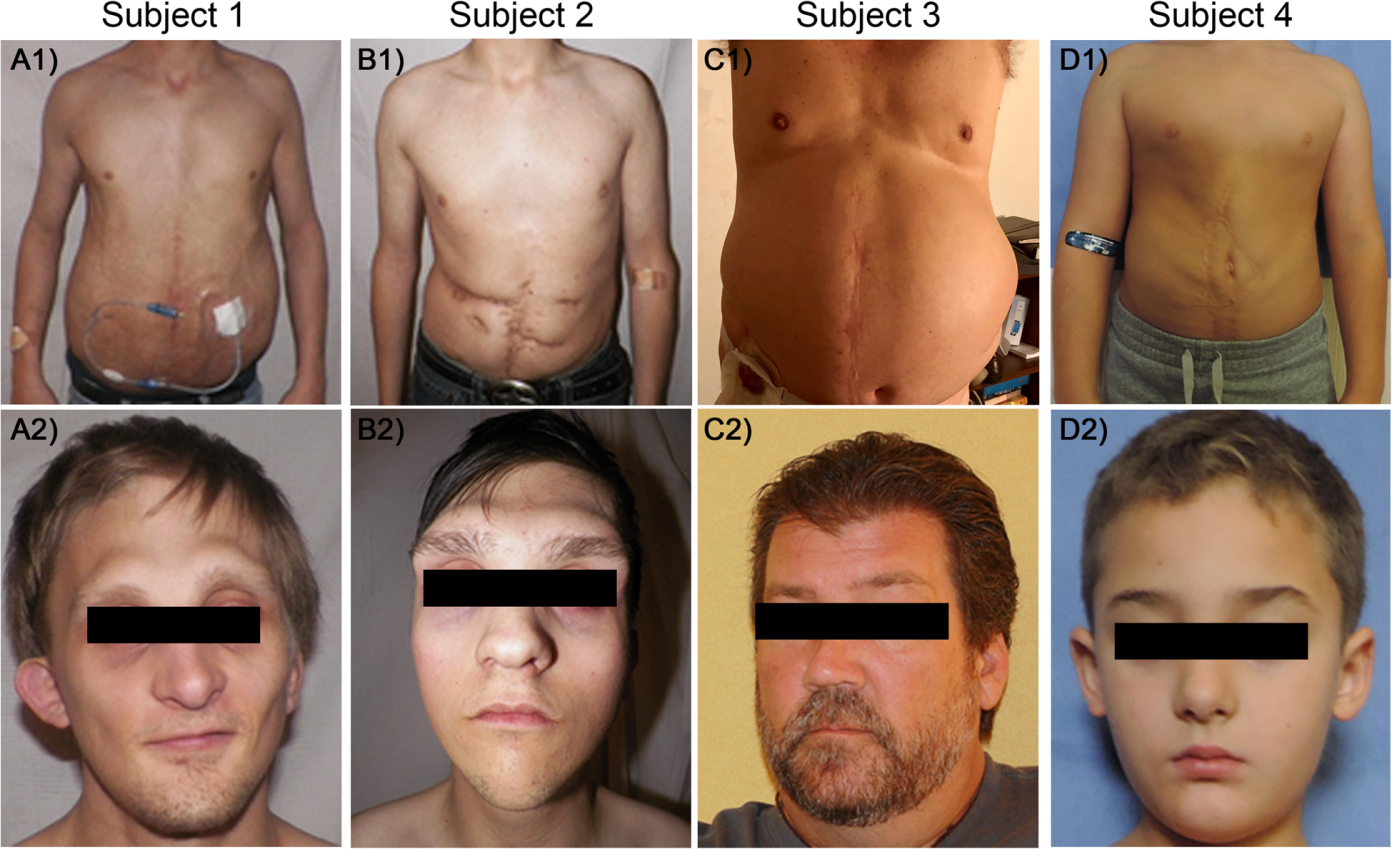
Thoraco-abdominal and Facial Photographs of 4 PBS affected individuals with *FLNA* mutations. **A1-A2**) Pedigree 1 Subject 1 (p.C2160R) has a RUBACE severity score of 22 and syndromic PBS with additional OPDSD features including prominent supraorbital ridge and micrognathia (**A2**). B1-B2) Pedigree 1 Subject 2 (p.c2160R) has a RUBACE severity score of 24 as well as syndromic PBS with OPDSD phenotypic features including prominent supraorbital ridge, micrognathia, facial asymmetry (**B2**). He additionally has Pierre Robin Sequence. **C1-C2**) Pedigree 2 Subject 3 (p.A1448V) has a RUBACE severity score of 14 (isolated PBS). No strong OPDSD craniofacial features are noted (**C2**). D1-D2) Pedigree 3 Subject 4 (p.G2236E) has a RUBACE severity score of 13 (isolated PBS). No strong OPDSD craniofacial features are noted (**D2**)

**Fig. 2:**
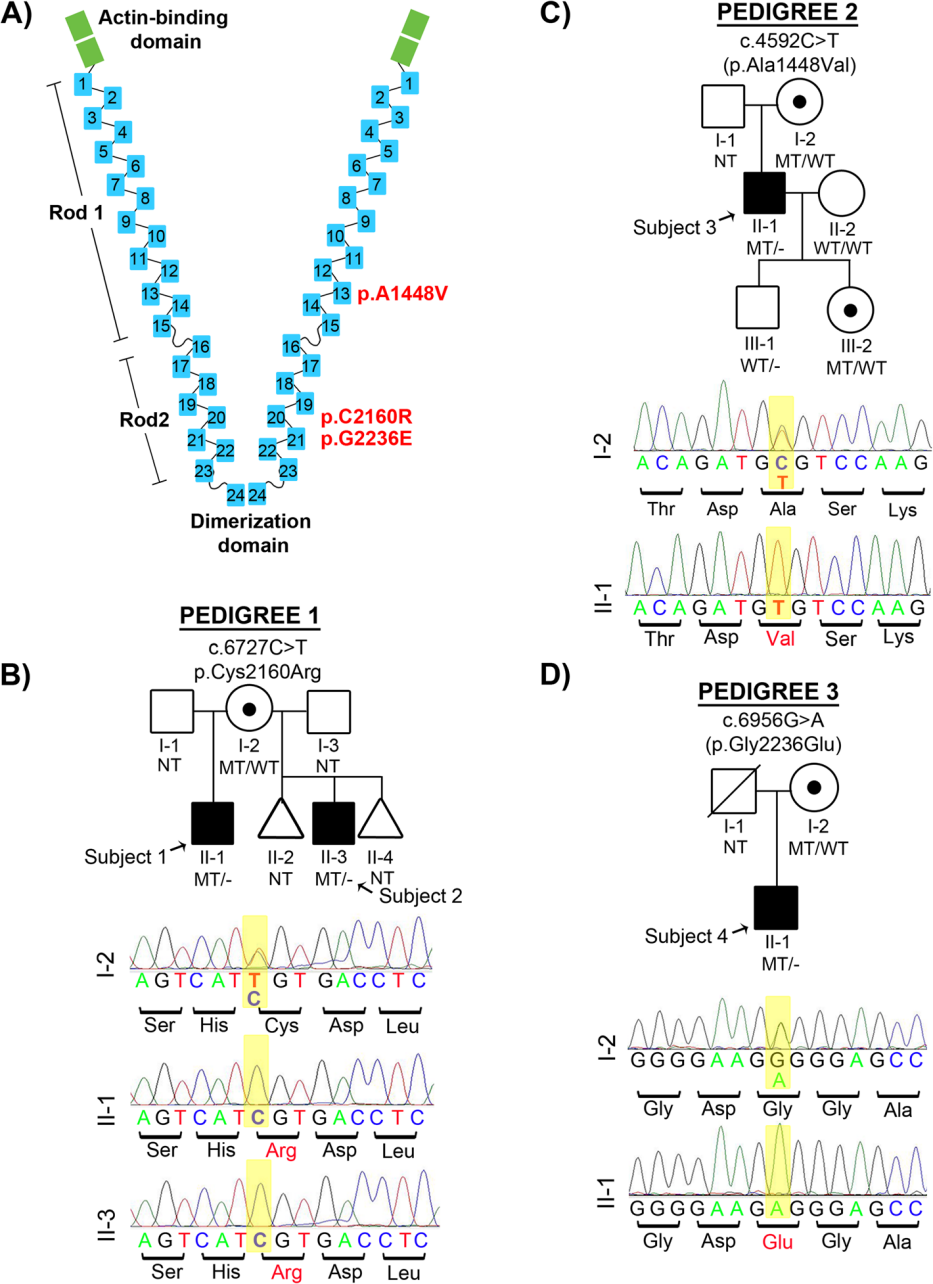
Pedigrees and *FLNA* mutations in PBS patients. **a** FLNA dimer is composed of an N-terminal actin binding domain (ABD), 24 immunoglobulin (Ig) repeats with two calpain-sensitive hinges separating the IgFLNA repeat domains into Rod 1 (IgFLNA1–15), Rod 2 (IgFLNA16–23), and C-terminal dimerization domain (IgFLNA24). Identified mutations are indicated in red. **b**-**d** Pedigrees and Sanger sequencing confirmation of identified mutations. Affected males are indicated in black squares, carrier females shown by circles with black dot. Genotyping results are shown by WT (wildtype), MT (mutant) or NT (not tested). Pedigree 1 is multiplex family with two affected half-brothers while Pedigrees 2 and 3 are of sporadic PBS cases. In all cases, co-segregation of identified mutation with PBS phenotype and maternal inheritance suggests X-linked recessive mode of inheritance

Subjects 1 and 2 (Fig. 1) are maternally shared PBS-affected half-brothers from Pedigree 1 (Fig. 2b) that present with syndromic PBS (PBS with previously undiagnosed OPDSD).

Subject 1 (Fig. 1 A1-A2) is a 29-year-old non-Hispanic white male with familial syndromic PBS (PBS + OPDSD) (RUBACE score 22). At 12 weeks gestation, he manifested bilateral severe hydronephrosis with renal dysplasia progressing to severe oligohydramnios which was treated with multiple fetal bladder aspirations. Born prematurely at 32 weeks gestation, he was initially ventilator dependent and had gastroesophageal reflux disease (GERD) requiring Nissen and gastrostomy tube. His urinary tract malformations included bilateral grade 4 VUR, urachal diverticulum, large capacity bladder, and urethral obstruction requiring genitourinary surgeries including neonatal vesicostomy, bilateral ureteral reimplantation, and urethral dilation. As an adult, he developed end stage renal disease and received a renal transplant. As he cannot urinate to completion, he empties his bladder by clean intermittent catheterization. His OPDSD features include distinct craniofacial and skeletal abnormalities including craniosynostosis with hydrocephalus requiring ventricular-atrial shunt, prominent supraorbital ridge, hypodontia, bilateral flaring of the anterior ribs, lumbar levoscoliosis, and bilateral genu valgum treated with distal femoral osteostomies (Supplementary Fig. 2: A1 and Table 1).

**Table 1:** Clinical features of Prune Belly Syndrome Subjects with FLNA mutations

PBS Subject		Subject 1		Subject 2		Subject 3		Subject 4	
Current Age, years		29 yrs		25 yrs		51 yrs		7 yrs	
Prenatal History (Gestational age at birth, weeks)		Oligohydramnios, multiple fetal bladder aspirations performed		Oligohydramnios		N/A (born at 40 weeks)		Oligohydramnios	
Stage of Life		Pediatric	Adult	Pediatric	Adult	Pediatric	Adult	Pediatric	Adult
PBS RUBACE Phenotype	Renal	Renal dysplasia	ESRD^b^ s/p^c^ renal transplant	Renal dysplasia requiring peritoneal dialysis	ESRD s/p cadaveric renal transplant	Obstructive uropathy requiring cutaneous ureterostomy	CKD^a^ stage 3	Normal renal function	
	Ureteral	Bilateral VUR with bilateral ureteral reimplantation, urethral obstruction requiring dilation		Right VUR treated with bilateral tapered ureteral reimplantation		Bilateral ureterovesical junction obstruction necessitating bilateral ureterostomies and bilateral ureteral reimplantation × 3, no VUR^d^ present		Bilateral VUR with spontaneous resolution	
	Bladder	Large bladder capacity with urachal diverticulum, vesicostomy and clean intermittent catheterization		Large capacity bladder, cutaneous continent vesicostomy, clean intermittent catheterization		Small capacity bladder (40-50 cc at 4.5 years of age) necessitating conduit diversion at age 6	Ileal conduit	Large capacity bladder s/p urachal diverticulectomy and clean intermittent catheterization	
	Abdominal Musculature	Severe laxity and wrinkling		Laxity	Mesh abdominal closure after Transplantation	Laxity	Right large direct and indirect inguinal hernia repair	Laxity	
	Cryptorchidism	Bilateral intraabdominal non-palpable testes (bilateral orchiopexy)		Bilateral inguinal testes (left orchiopexy and right orchiectomy)		Bilateral undescended testes (bilateral orchiopexy)	Right orchiopexy; recurrent large hydroceles	Bilateral non-palpable testes (bilateral orchiopexy)	
	Extra-genitourinary: Gastrointestinal	Nissen and Gastrostomy tube for GERD^e^, Constipation		Malrotation, duodenal perforation, Nissen and gastrostomy tube for GERD, Constipation			Constipation	Constipation	
	Extra-genitourinary: Respiratory	Ventilator dependent at birth	Reactive airway disease	Ventilator dependent in infancy		Right Upper lobe pneumonia in first month of life	Normal	Normal	
	PBS RUBACE Severity Score (range 0–31) and PBS category	22, Syndromic PBS		24, Syndromic PBS		14, Isolated PBS		13, Isolated PBS	
OPDSD Phenotypes	Craniofacial	Craniosynostosis, Skull base sclerosis, Prominent supraorbital ridge, full cheeks, hypodontia, micrognathia		Prominent supraorbital ridge, down slanting palpebral fissures, proptosis, ocular hypertelorism, hypodontia, micrognathia and facial asymmetry (Pierre Robin Sequence)		Normal		Normal	
	Deafness	Absent		Congenital hearing loss bilaterally (hearing aids), recurrent otitis media and PET		Absent		Absent	
	Cleft Palate	Absent		Yes, Bilaterally surgically corrected		High arched palate		Absent	
	Thorax	Pectus carinum, Irregular ribs (flaring of the anterior ribs bilaterally)		Pectus carinum, Irregular ribs (absent T12 ribs bilaterally)		Normal		Normal	
	Heart	Atrial Septal Defect		Atrial Septal Defect		Normal		Normal	
	Omphalocele	Absent		Absent		Absent		Absent	
	Scoliosis	Lumbar Levoscoliosis, non-surgical		T5-L4 Kyphoscoliosis s/p Posterior Spinal Fusion and L3-pelvis fusion (residual 38° thoracic levoscoliosis and 88° thoracolumbar kyphosis)		Mild, non-surgical		Not present	
	Limbs/Digits	Bilateral genu vaigum S/P bilateral distal femoral osteostomies, Short Achilles tendon, Broad thumbs, 2 nails on right 3rd toe, Hammer shaped toe, wide spaced toes, Hypoplastic distal phalanges		Short proximally placed thumbs, hypoplastic distal phalanges, Short Achilles tendon, hypoplasia of the great toe, long second toe		Hypoplastic distal phalanges, hypoplasia of the great toe		Hypoplastic distal phalanges, hypoplasia of the great toe	
	CNS	Hydrocephalus S/P shunt X 2, Seizures		Normal		Normal		Normal	
	Mentation	ADHD^f^ treated with concerta		Moderate Developmental Delay		Normal		Developmental Delay	
	Other Anomalies	Hypotonia, thrombocytopenia				None		None	

^a^ CKD (Chronic kidney disease)

^b^ ESRD (End stage renal disease)

^c^ S/P (status post)

^d^ VUR (vesicoureteral reflux)

^e^ GERD (gastroesophageal reflux)

^f^ ADHD (attention-deficit/hyperactivity disorder)

Subject 2 (Fig. 1 B1-B2) is a 25-year-old non-Hispanic white male with familial syndromic PBS (PBS + OPDSD) (RUBACE score 24). At 16 weeks gestation, a grossly enlarged bladder with oligohydramnios was diagnosed. Born prematurely at 32 weeks gestation, he was initially ventilator dependent for 2 months; episodes of aspiration pneumonia and gastroesophageal reflux disease (GERD) mandated Nissen and gastrostomy tube. Genitourinary anomalies included bilateral renal dysplasia necessitating neonatal temporary peritoneal dialysis, large capacity bladder and megaureters requiring neonatal vesicostomy and later partial cystectomy with bilateral tapered ureteral reimplantation, catheterizable continent vesicostomy and bilateral intraabdominal cryptorchidism. Gastrointestinal phenotypes include intestinal malrotation requiring LADD procedure and temporary jejunostomy for duodenal perforation. As an adult, he developed end stage renal disease requiring renal transplantation, which warranted a mesh abdominal wall reconstruction due to his severe degree of abdominal wall weakness at the time of transplantation. Like his half-brother, he empties his bladder by clean intermittent catheterization. His OPDSD features include prominent supraorbital ridge, down slanting palpebral fissures, proptosis, ocular hypertelorism, hypodontia, facial asymmetry, conductive hearing loss, developmental delay, cleft palate, bilateral absence of the T12 ribs, “tree-frog” feet, bilateral pars defects and spina bifida at L5, and severe scoliosis requiring multiple surgical interventions (Supplementary Fig. 2: B1-B2 and Table 1).

Subject 3 (Fig. 1 C1-C2) is a 51-year-old non-Hispanic white male from Pedigree 2 (Fig. 2c) with sporadic isolated PBS (RUBACE score 14). As a term infant, his urinary tract was devastated, presenting with bilateral ureteral obstruction with acute kidney injury requiring > 7 urinary tract reconstructions and diversions (neonatal bilateral cutaneous ureterostomies, 3 ureteral reimplantations, and ileal conduit diversion at age 6 yrs). Now, he remains with an ileal conduit, has required partial nephrectomy, and has recurrent kidney stones with renal insufficiency (CKD3, GFR 30 ml/min/1.73m^2^). Despite many surgeries, his abdominal wall is lax requiring recurrent hydrocele and inguinal hernia repairs after childhood orchiopexies. Phenotypic features of OPDSD in Subject 3 include mild scoliosis, a high arching palate and broad shortened distal phalanges but prominent craniofacial abnormalities are not noted (Supplementary Fig. 2: C1-C2 and Table 1).

Subject 4 (Fig. 1 D1-D2) is a 7-year-old non-Hispanic mixed race male from Pedigree 3 (Fig. 2d) with sporadic isolated PBS (RUBACE score 13). Prenatal diagnosis of obstructive uropathy with hydronephrosis who was born term but small for gestational age and did not require intubation/ventilation. Postnatally, he had bilateral pelvocaliectasis with bilateral grade 2 VUR and a large capacity bladder with urachal diverticulum. He has had urachal diverticulectomy, abdominoplasty, and bilateral orchiopexy for intraabdominal testes. Currently he has normal renal function and no VUR but empties his bladder by clean intermittent catheterization. Phenotypic features of OPDSD in Subject 4 include developmental delay, short and broadened distal phalanges, short and broad halluces, but prominent craniofacial abnormalities are not noted (Supplementary Fig. 2: D1-D2 and Table 1).

### WES identifies mutations in *FLNA* in PBS affected patients

We first obtained DNA from the two affected maternally shared half-brothers in the multiplex family (Fig. 2b, Pedigree 1 II-1 and II-3) and performed paired end whole exome sequencing (WES) from peripheral blood DNA on both. Details on WES metrics and variant analysis are provided in Supplementary Table 1. Filtering of the variant call files for variants that met our filtering criteria (coding or impact a splice site, not reported in public databases) and shared by both half-brothers revealed *FLNA* c.6727C > T (GeneBank: NM_001110556.1); p.Cys2160Arg variant on chromosome X as the only recessive variant shared by both half-brothers (Supplementary Table 1). The alternative allele was identified in 100% of WES reads at this position in both affected male individuals and is not reported in ExAC or gnomAD. Sanger sequencing confirmed that both PBS half-brothers are hemizygous for this variant and their mother (Fig. 2b, Pedigree 1, I-2) is a heterozygous carrier with 95:5 ratio of skewed X-chromosome inactivation by androgen receptor methylation assay, suggesting a protective mechanism against a deleterious mutation on one X-chromosome and presumably preferred expression of the wild type allele (Supplementary Fig. 1). Although not previously clinically diagnosed, the half-brothers manifest PBS with Otopalatodigital (OPD) spectrum disorder (OPDSD) while their mother has no PBS features but has mild OPDSD phenotypic manifestations (Table 1 and Supplementary Fig. 1) [2, 44]. Although there is unconfirmed maternal family history of PBS in earlier generations (expanded pedigree in Supplementary Fig. 1), none are living to test for presence of the variant. Biallelic autosomal variants common to both brothers that met the filtering criteria were not found in this family and because a recessive or X-linked recessive mode of inheritance is hypothesized for PBS, *FLNA* emerged as the most likely candidate gene in this multiplex PBS kindred. The p.C2160R mutation within FLNA Rod 2 (Fig. 2A) affects a highly conserved residue of the immunoglobulin repeat 20 of FLNA (IgFLNA20), which is within the integrin interaction domain (Fig. 4a). Integrins form heterodimers (ITGα/ITGβ) that have extracellular ligand-binding loops and a C-terminal cytoplasmic interactive tail. FLNA Rod 2 binds the cytoplasmic tail of β-integrins, mediating cell contraction and remodeling of collagen matrices (in the case of FLNA/ITGβ1) [45]. In response to mechanical force, ITGβ1 recruits both FLNA and actin to ITGβ1-containing membrane focal adhesions [46]. Taken together, these data suggest that the identified PBS FLNA variant in the two half-brothers has a potential functional impact on the mechanosensing properties of FLNA through its interaction with integrin beta tails.

To further investigate whether *FLNA* variants cause PBS in other unrelated individuals, we searched our WES database of sporadic and familial PBS patients and found variants in *FLNA* in four sporadic PBS-affected individuals (Supplementary Table 2). Upon Sanger sequencing validation and inheritance testing, two of these four variants (p.Arg24Leu and p.Gly2138Cys) did not segregate with the phenotype in the family and were therefore not pursued for further investigation. Furthermore, the p.Gly2138Cys mutation did not meet our WES filtering criteria as it was reported in ExAC in three male individuals (MAF =0.00009) suggesting that this mutation is not causal for PBS.

However, the remaining two variants were in two unrelated sporadic isolated PBS male cases without OPDSD (Fig. 2c and d, Pedigree 2 II-1 and Pedigree 3 II-1). They harbor hemizygous non-synonymous, novel mutations in *FLNA* that fit our filtering criteria– c.4952 C > T (GeneBank: NM_001110556.1); p.Ala1448Val (Subject 3) and c.5966 G > A (GeneBank: NM_001110556.1); p.Gly2236Glu (Subject 4). Details on WES data are available in Supplementary Table 1. The variants identified in the two sporadic individuals affect highly conserved residues of FLNA and are not observed in the ExAC database. In all cases, the identified *FLNA* variants are maternally inherited, further supporting an X-linked recessive mode of inheritance. In summary, we identified three unreported missense point mutations across four individuals in *FLNA* as causal for PBS (Figs. 1 and 2).

### FLNA is expressed in bladder smooth muscle

**Fig. 3:**
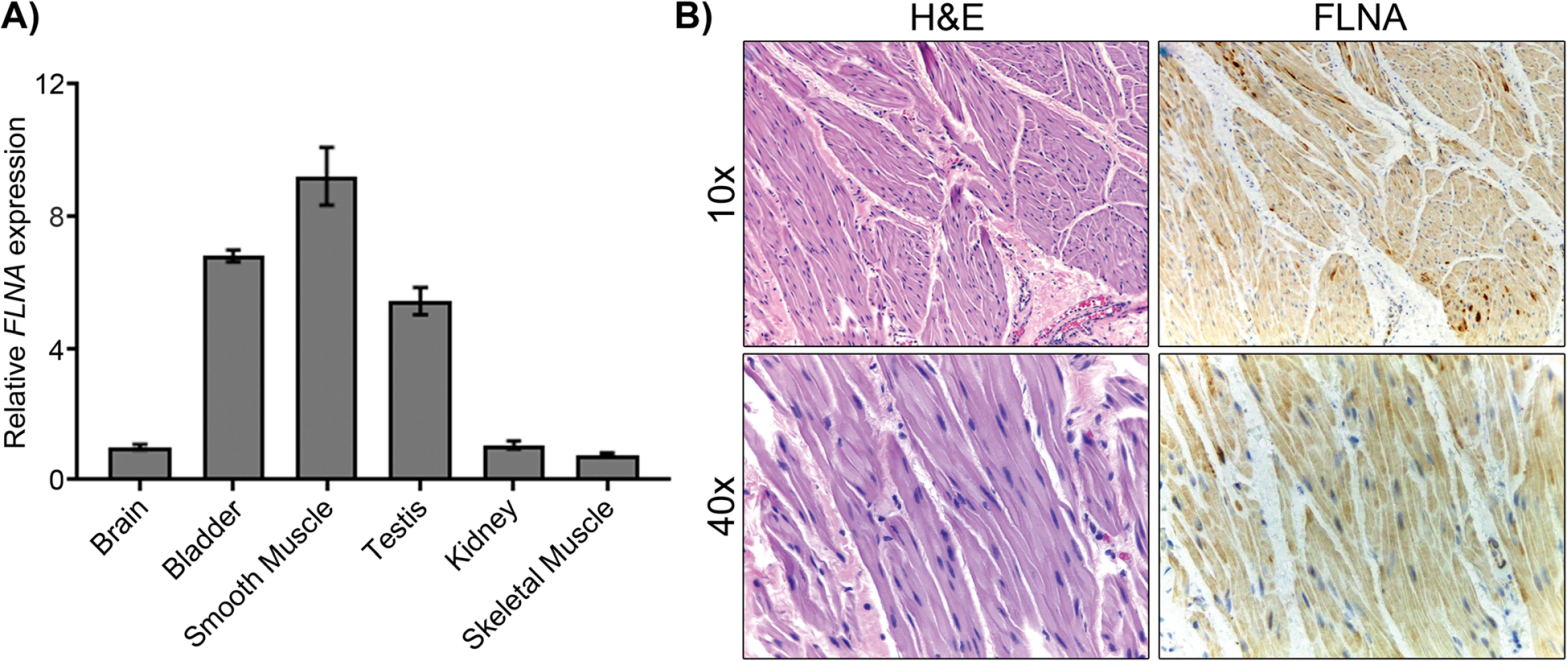
FLNA is expressed in human bladder smooth muscle. **a** qPCR across adult human tissues. *FLNA* expression normalized to *GAPDH* and relative to brain. **b** IHC from pediatric normal human bladder shows cytoplasmic and some nuclear smooth muscle expression of FLNA

To assess the relationship between the PBS phenotype and mutant FLNA, we examined normal FLNA spatiotemporal expression. We first consulted publicly available expression databases including GenePaint and EurexpressII, transcriptome-wide compendiums of mouse in situ hybridizations during fetal life. *Flna* is highly expressed in the developing bladder detrusor, ureter, abdominal wall musculature, urogenital sinus mesenchyme, intestinal smooth muscle inner circular layer, discrete cardiac regions, alveolar buds of the lung as well as the sternum of the embryonic day 14.5 wild type mouse. These expression patterns correlate with the cardinal features and the extra-genitourinary manifestations of PBS [2, 3]. To compare spatial expression of *FLNA* in humans, we performed qPCR on RNA isolated from normal pooled adult human donor tissues and found *FLNA* to be highly expressed in smooth muscle-containing organs with strong enrichment in the small intestines and urinary bladder (Fig. 3a). To assess for age -related differences in expression, four pediatric normal bladder biopsies (without PBS) were evaluated by FLNA immunohistochemistry, which revealed predominant expression of FLNA within bundles of smooth muscle cells but not in intervening connective tissue (Fig. 3b). Although we did not have bladder biopsies from the four PBS males described in this report, FLNA bladder IHC on 3 other PBS cases of undiagnosed molecular cause revealed no significant change in FLNA protein immunostaining (data not shown), which is consistent with gain of function FLNA mutations that do not disturb FLNA protein quantity (see below in Discussion)*. FLNA* expression in smooth muscle tissues, particularly the bladder detrusor, in both mice and humans is consistent with the phenotypes observed in PBS and further supports it as a novel PBS causal gene.

### Mutations in FLNA affect binding to integrin proteins

**Fig. 4:**
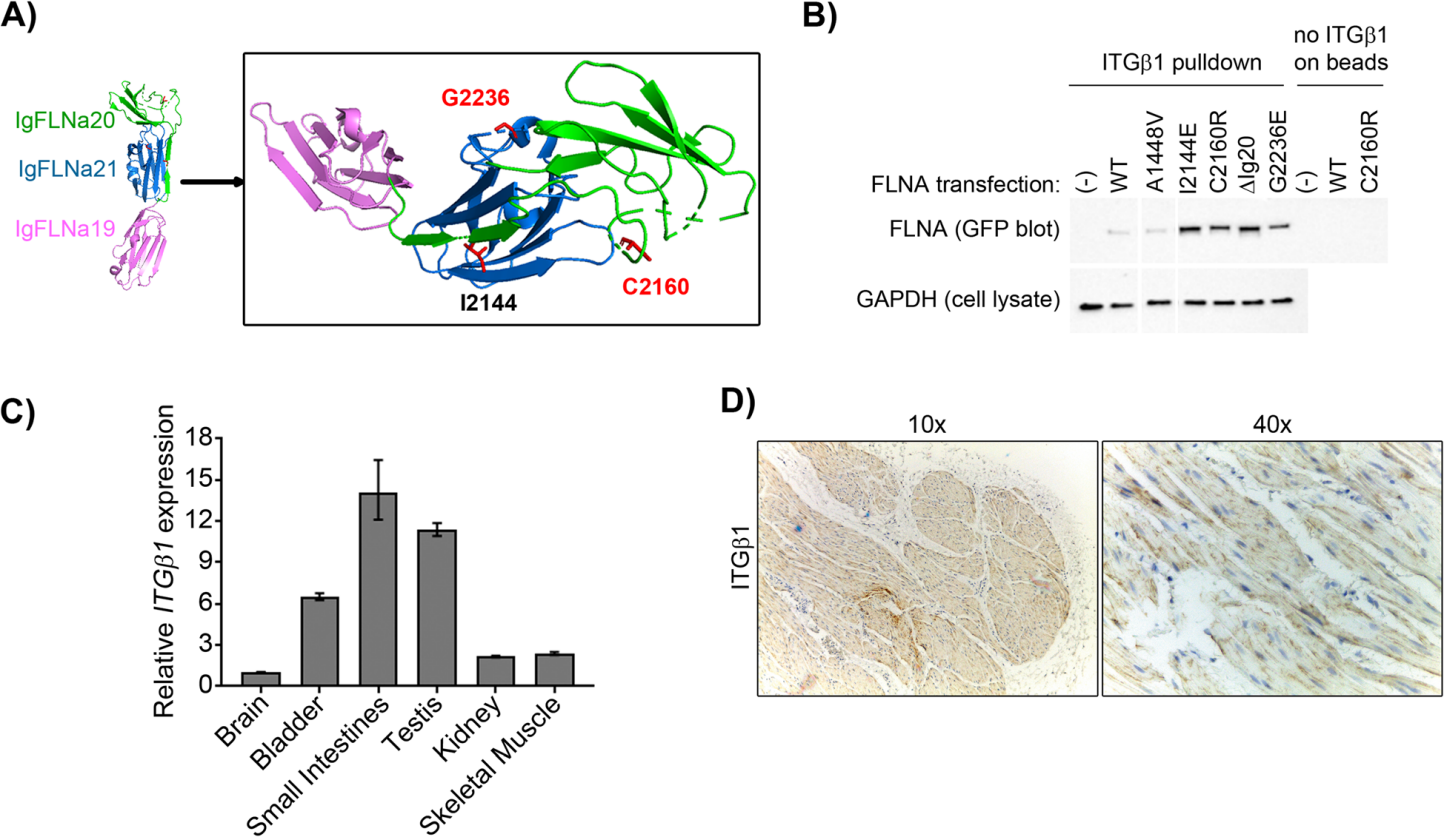
PBS FLNA mutations disrupt binding to integrin. **a** PyMol 3D protein standard cartoon of wildtype IgFLNA repeats 19 (magenta), 20 (green), and 21 (blue) showing the auto-inhibitory IgFLNA20 β-strand A on the β-strand C/D face of IgFLNA21. Shown in red are C2160 and G2236, the locations of the two FLNA residues in Ig20 and Ig21 mutated in PBS (PDB code 2J3S). The position of the IgFLNA20 β-strand A residue I2144 is shown in black; although it has not been reported mutated in humans, the I2144E mutation has been shown to lead to increased FLNA-ITGβ binding. The FLNA PBS mutation p.A1448V in Ig13 is not shown. **b** Pulldown assay showing that p.C2160R and p.G2236E mutations enhance binding to ITGβ1 similarly to engineered positive controls p.I2144E and p.ΔIg20. In contrast, p.A1448V binds ITGβ1 similar to WT. CHO cells were transfected with full length FLNA and bound to ITGβ1 tails on beads. **c** qPCR across adult human tissues shows *ITGB1* expression is highest in small intestines but also strong bladder expression. *ITGB1* expression normalized to *GAPDH* and relative to brain. **d** IHC from pediatric normal human bladder shows plasma membrane smooth muscle expression of ITGβ1

In order to assess the functional impact of the PBS-associated FLNA mutations, we interrogated the FLNA-integrin binding interaction, a well characterized interaction important for cell migration, polarity, and contraction [47–50]. While both ITGβ1 and 7 have been demonstrated to bind to IgFLNA21, unlike ITGβ7, ITGβ1 is highly expressed in mouse bladder and small intestinal smooth muscle as well as abdominal wall skeletal muscle tissues (GenePaint, EurexpressII) [47, 49]. Further, ITGβ1 specifically localizes to the plasma membrane of smooth muscle cells from human pediatric normal bladder tissue (Fig. 4c and d). Thus, to investigate the functional consequences of the identified *FLNA* mutations on the interaction between FLNA and ITGβ1, CHO (Chinese hamster ovary) cells were transfected with full length FLNA plasmids carrying the candidate PBS mutations, and cell lysates were used for pull down assays with immobilized β-integrin tails as described [43, 47, 49]. The previously studied artificial FLNA mutations I2144E (in IgFLNA20) and ΔIg20 are not PBS-related, but have been engineered to demonstrate the auto-inhibitory mechanism of IgFLNA20 on IgFLNA21 which normally blocks ITGβ1 binding to FLNA [47, 49] (Fig. 4a). ΔIg20 and I2144E exhibit enhanced FLNA binding to β-integrin tails resulting from the “open” configuration of the C/D binding face of IgFLNA21, allowing enhanced FLNA/integrin interactions without regulation from a force-induced signal [48]. These mutations were included in this study as positive controls for disruption of IgFLNA20–21 interactions. As expected, I2144E, which introduces a large polar acidic residue into IgFLNA20, strongly enhances binding to ITGβ1. Similarly, we observed a strong increase in binding of FLNA to ITGβ1 tails with the PBS FLNA C2160R mutation and to a lesser extent in the G2236E mutant when compared to WT. There was no change observed with the FLNA A1448V mutation (Fig. 4b). Comparable results were obtained in a direct binding assay using purified GST-FLNA Ig19–21 proteins (data not shown).

## Discussion

In this report, we identify four surviving males (two with familial syndromic PBS + OPDSD and two with sporadic isolated PBS) to harbor three different mutations in the X-chromosome gene, *FLNA*, thereby expanding the phenotypic spectrum attributed to the filaminopathies. FLNA belongs to a family composed of three paralogues including *Filamin A* (*FLNA*), *Filamin B* (*FLNB*) and *Filamin C* (*FLNC*). Structurally, the filamins (FLN) form V-shaped homodimers, resulting in orthogonal crosslinking of F-actin. FLNs can be cleaved at two hinge sites into Rod1 (IgFLN1-IgFLN15) with multiple actin-binding domains (ABD) (including a high avidity F-actin binding domain in IgFLNA9–15) and Rod 2 (IgFLN16–IgFLN23) which does not bind actin but is stretch-sensing and binds many regulatory proteins [51]. In addition, each monomer of FLN also contains an N-terminal ABD and a C-terminal dimerization domain at Ig24 (Fig. 2a) [51]. The versatility of filamin functions, diversity of expression patterns and heterogeneity of binding partners is reflected in the spectrum of human disease phenotypes attributed to the filaminopathies. Deleterious mutations in *FLNA, FLNB* and *FLNC* cause malformations of the brain, cranium, face, skin, viscera, skeleton, heart, vasculature and muscle [52–56].

**Fig. 5:**
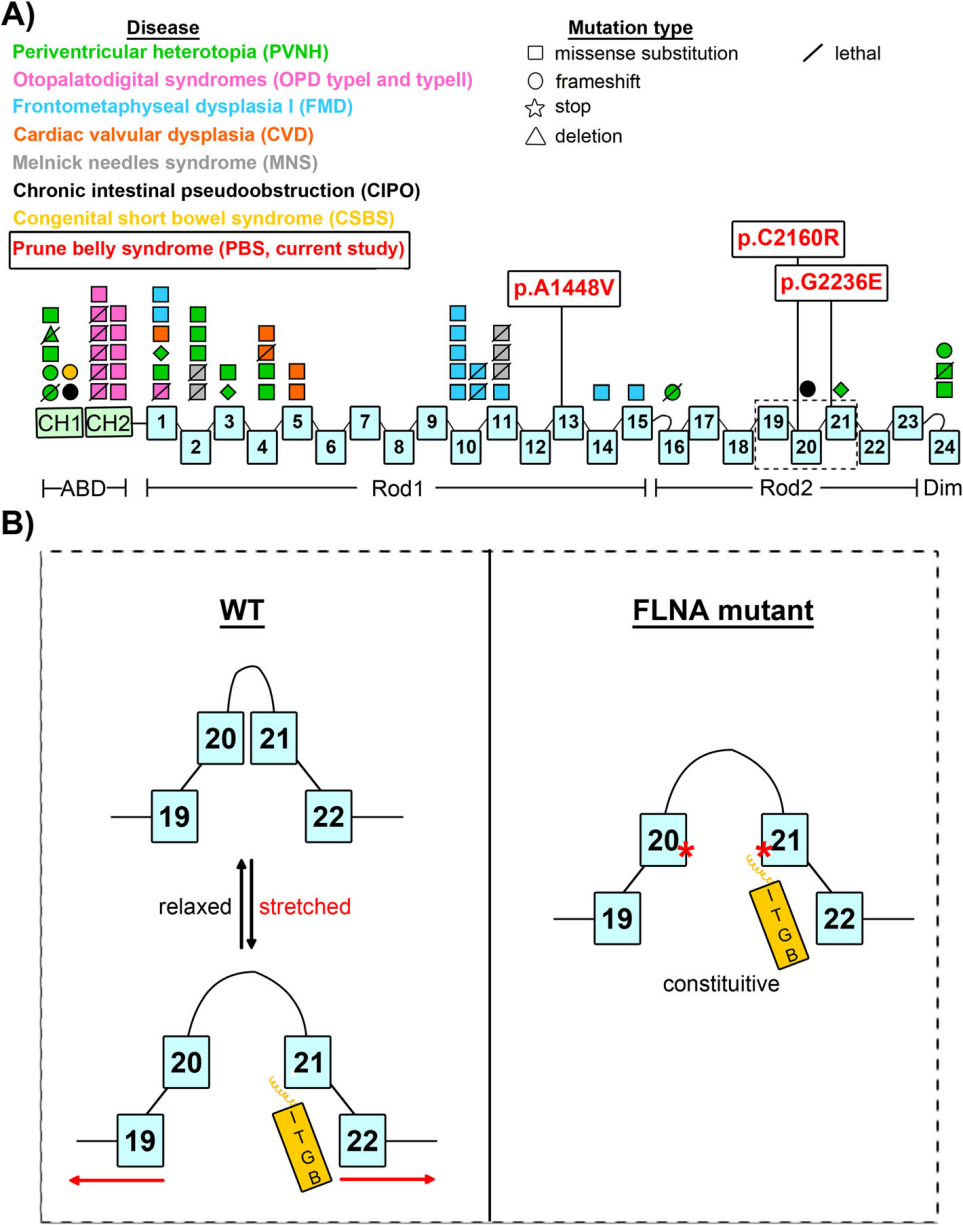
Human Disease Causing Mutations in *FLNA* in Surviving and Non-surviving Males. **a** *FLNA* disease-causing mutations cluster in ‘hot spots.’ PVNH mutations are largely found in the actin binding domain (ABD) while OPD spectrum disorders (including OPD, FMD and MNS) cluster in IgFLNA10. We identified 3 novel PBS variants: A1448V in IgFLNA13 (Rod 1), C2160R in IgFLNA20 and G2236E in IgFLNA21 (Rod 2). **b** Model for FLNA interaction with integrins. In a relaxed state, there is an auto-inhibitory interaction between Ig20–21 that masks the integrin binding site on Ig21. Normally, mechanical stretching can cause exposure of the integrin binding site on Ig21. Mutations in Ig19–21 result in loss of FLNA function as a stretch sensor and disruption of the auto-inhibitory interaction between Ig20–21 causing constitutive exposure of the integrin binding site on Ig21 and thereby enhanced binding to integrins

Unlike *FLNB* and *FLNC* which are autosomal genes, *FLNA* is a dominant X-linked gene with a wide but regionally intense expression pattern. FLNA mutations are broadly described as either loss-of-function (LOF), with reduced or absent FLNA expression, or gain-of-function (GOF), with normal expression of a full length in-frame mutant pathogenic FLNA protein [57]. FLNA mutations cause a wide spectrum of phenotypically overlapping genetic diseases [58]. The classic OPDSDs are osteochondrodysplasias including OPD1, OPD2, frontometaphyseal dysplasia (FMD), MNS, and terminal osseous dysplasia with pigmentary defects (TOD) [42]. Beyond the OPDSDs, *FLNA* variants have also been shown to cause XCVD, PVNH1 and PVNH4, childhood interstitial lung disease (ChILD), structural cardiac and aortic anomalies, thoracic aortic aneurysms (TAA), chronic intestinal pseudo-obstruction (CIPO), and congenital short bowel syndrome (CSBS) (Fig. 5a) [57, 59–71]. Typically, PVNH, XCVD, CIPO and CSBS are thought to be caused by LOF mutations while OPDSDs are caused by GOF *FLNA* mutations.

*FLNA* mutations in males is often lethal, however, a small subset with missense *FLNA* mutations have been reported to survive beyond the first weeks of postnatal life. (Fig. 5a) [41, 67, 72]. Exceedingly rarer are adult surviving males with *FLNA* mutations. Herein, we document three additional surviving adults with *FLNA* mutations and draw focus upon the genitourinary phenotypes caused by *FLNA* mutation. Past publications have broadly noted “obstructive uropathy” or “genitourinary abnormalities” in rare male patients who died neonatally with *FLNA* mutations. In 1987, prune belly sequence was observed in a MNS patient not sequenced for *FLNA* [73]. More recently, four males with lethal MNS and *FLNA* exon 22 mutations disrupting Ig10 have been described with genitourinary abnormalities including omphalocele, megacystis, and/or “prune belly-like” phenotype of abdominal wall laxity [58, 74, 75]. A lethal form of FMD from *FLNA* exon 22 mutation was noted in a male with distended abdomen, megaureters and hydronephrosis [76]. Additionally, a 2009 report of an Xq28 duplication (which includes *FLNA*) described a kindred with several males who died as infants, one of which was diagnosed with prune belly syndrome and another with a grossly distended bladder after birth [77]. Overall, there is not a clear genotype-phenotype correlation when these old published cases are added to our series, other than the fact that most MNS have FLNA exon 22 mutations within Ig10. As FLNA Ig10 is quite remote from the FLNA Ig19–21 domain, it is biologically unclear how missense mutations in Ig10 yield the obstructive uropathy phenotype. As these genotypic differences exist and since PBS due to deleterious *FLNA* mutations is seen with or without OPDSD, we prefer to segregate our PBS phenotype from the MNS phenotype, as depicted in Fig. 5a.

FLNA is a critical intracellular connecting protein between the cytoskeleton and transmembrane complexes that bind to extracellular matrix. At the protein level, the FLNA:integrin interaction is highly regulated, as in vitro excessive integrin binding of FLNA prevents efficient actin remodeling and cell motility [78]. Specifically, the FLNA repeat domains Ig20 and Ig21 are critical for this regulation, functioning as a stretch mechanosensor [79, 80]. The binding site for β-integrin tails is on IgFLNA21 but when FLNA is not stretched, this site is blocked by the β-strand A of IgFLNA20, suggesting an auto-inhibitory mechanism regulating FLNA:integrin binding [48]. A mechanism for up-regulation of FLNA integrin binding is force-induced mechanical stretching of FLNA, permitting integrin binding. When FLNA is stretched, IgFLNA20 is disengaged from IgFLNA21 thereby exposing the IgFLNA21 C/D face for binding of integrins to IgFLNA21 and stabilizing the stretched FLNA conformation. Once force is released, the IgFLNA20 B-strand refolds back onto IgFLNA21, displacing integrin [45, 46, 81–85]. Two of our detected PBS IgFLNA19–21 variants alter binding of FLNA to β-integrin tails. The PBS mutations C2160R (in IgFLNA20) and G2236E (in IgFLNA21) are both substitutions of small uncharged residues to large charged residues with multiple side chains. The newly introduced, large charged residues likely significantly contribute to the disruption of the IgFLNA20–21 interface, presumably stabilizing the exposure of the integrin binding site on IgFLNA21 leading to enhanced binding of ITGβ1 tails similarly to the engineered I2144E and ΔIg20 mutations (Fig. 5b). Altered transmembrane receptor binding to FLNA and ligand-independent phosphorylation of FLNA has been reported in the IgFLNA20 P2204L mutation found in FMD [86]. Improper mechanosensing properties of FLNA likely leads to apoptosis in force-loaded cells [87].

The Rod1 A1448V variant is located in the N-terminal region of Ig13, which has not been reported to bind integrins. Unlike the ligand binding IgFLNA19 and IgFLNA21 Class A repeats, Ig13 is a member of the class D Ig repeats (which includes IgFLNA10). IgFLNA10 has been crystalized and functional implications of mutations causal for MNS have been assessed such that the p.A1188T and p.S1199L MNS mutations are predicted to alter hydrophobic packing of IgFLNA10 and possibly impact FLNA stability [88]. Together, these data suggest that C2160R and G2236E may indeed cause disease via disrupted integrin-dependent function of FLNA while the A1448V substitution may affect an integrin-independent function of FLNA.

FLNA is critical in embryonic development. 100% of hemizygous male mice null for *Flna* die by E14.5 with widespread hemorrhage, incomplete heart outflow tract septation (common arterial trunk), ventricular septal defects, and mitral valve dysplasia [89, 90]. Coarse dilated blood vessels within many tissues led to organ malformations and angiogenesis was disrupted by aberrant adherens junctions in endothelial cells [89]. Additional midline malformations in these male mice include non-fused split sternum, umbilical hernia and cleft palate [90]. *Flna* binds actin-nucleating *Formin 2 (Fmn2)* and *Flna + Fmn2* null mice exhibit microcephaly, thoracoabdominal schisis, thinned ventral body wall (muscle, ribs, and sternum) and shortened gut lengths when compared to WT embryos, partially due to fewer proliferating mesenchymal cells in the sternum and ribcage [91]. The inbred Long Evans orl rat has spontaneous cryptorchidism secondary to abnormal formation of the gubernaculum, the cord-like ventral abdominal wall appendage that normally drags the testis into the scrotum during embryonic life. The orl rat has altered *Flna* expression in its gubernaculum, which is comprised of a core of mesenchymal cells with associated extracellular matrix and localized striated muscle [92]. These data stress the essential role of *Flna* for normal cardiac, vascular, skeletal, abdominal wall, gubernaculum, and gastrointestinal development during embryogenesis. Beyond embryonic life, conditional deletion of *Flna* in adult murine smooth muscle lineages such as vascular smooth muscle cells (VSMCs) induces hypertrophic remodeling of the carotid artery and aorta [93]. Zhu et al observed that when rabbit aortic VSMCs are overproliferating or actively migrating, FLN is expressed at elevated levels. Conversely, when expression of FLN is decreased, VSMCs return to the “contractile” phenotype, suggesting a key role in VSMCs phenotype switching [94]. In cultured cells, Flna has been shown to interact with Mkl1 and promote a Srf-dependent smooth muscle transcriptional program [95].

Our PBS cases highlight the role of *FLNA* in muscle development and function. In humans, several *FLNA* disease phenotypes manifest smooth muscle dysfunction (leiomyopathy), including TAA, CIPO and CSBS. Recent work by Jenkins et al. has explored why some males with 5′ mutations phenotypically only manifest CIPO. They identified tissue-specific differential expression of two *FLNA* transcripts and suggest the longer protein isoform (ATG^+ 1^) is crucial for smooth muscle development [57]. Alternatively, whether there is a *FLNA* mutational hotspot for leiomyopathies is not clear. In support of this concept, a familial case of CIPO and intestinal malrotation in two affected brothers was shown to be caused by a 4 bp deletion of *FLNA* exon 40 causing an in-frame exon skipping affecting FLNAIg20 [67]. Very few mutations have been identified in the highly studied IgFLNA19–21 region, however, this triplicate domain of FLNA is not only important for binding of many interacting partners including integrins, but potentially for FLNA function unique to smooth muscle cells and now PBS [39, 40, 64, 96, 97].

The three cardinal features of PBS can thus be explained by deranged FLNA signaling. However, PBS is a complex phenotype, ranging from neonatal lethality to mild manifestations. PBS lethality is most commonly associated with in utero oligohydramnios from low volume of fetal urine expelled from the urinary system. This low urine volume may be due to poor urine production by a malformed fetal kidney and/or improper bladder and ureteral emptying. Oligohydramnios is also associated with abnormal lung development (bronchopulmonary dysplasia) often associated with respiratory death. In mice, Flna is expressed in the fetal alveoli and airway smooth muscle cells, fetal glomeruli and urinary tract urothelium and smooth muscle cells – all of which are crucial for postnatal life.

At this point, many cases of PBS remain genetically undefined. Other plausible genes implicated in the pathogenesis of PBS include variants in *CHRM3* (the muscarinic cholinergic receptor M3 responsible for bladder smooth muscle contraction), *ACTA2* and *ACTG2* (two actin genes expressed in smooth muscle), *HNF1β* (the embryonic transcription factor hepatocyte nuclear factor *1β)* and *STIM1* (the stromal interaction molecule 1 which has calcium channel regulatory activity). Variants in these genes only explain single cases or one independent consanguineous family [32–36, 98, 99]. Our WES data has not identified exonic mutations in *CHRM3*, *HNF1β* or *STIM1* in PBS patients, although we have found one individual with a previously unreported heterozygous variant in *ACTA2* and two individuals with previously unreported heterozygous mutations in *ACTG2.* Whether the reported actin mutations directly interfere with FLNA binding is unknown. The long term implications of making a molecular diagnosis in cases of PBS are significant, as PBS patients harboring a *FLNA* or *ACTA2* mutation may need to adopt surveillance strategies for cardiovascular disease such as moya-moya or TAA [69, 71, 100]. Overall, this suggests that PBS is not monogenic, but may be caused by mutations in multiple genes and that other causal genes remain to be identified [32].

Our study does have limitations. Our PBS study did not include *FLNA* WES in perinatal PBS deceased cases. Our cohort of surviving patients with PBS likely induces a survivorship bias, thus limiting and underestimating the number of PBS cases (lethal or living) with FLNA mutations. We did not demonstrate any recurrent *FLNA* mutations in PBS in our small cohort. Lastly, our study suffers from the lack of additional tissues or patient derived cells for further correlative testing and the lack of a PBS mouse model. Nevertheless, our data is compelling.

## Conclusions

In summary, we have identified three hemizygous mutations in *FLNA* in one multiplex kindred and two sporadic PBS males with or without OPDSD phenotypes who have survived even to adulthood. This report highlights the role of *FLNA* in rhabdomyopathy, cardiomyopathy and leiomyopathy. It also represents the first proposed PBS candidate gene to support an X-linked recessive mode of inheritance and the first candidate gene identified in both familial and unrelated sporadic individuals, representing the largest number of cases with mutations in the same gene as causal for the congenital myopathy PBS.

## Supplementary information

**Additional file 1: Table S1.** Whole exome sequencing metrics and variant calling. **Figure S1.** X-linked recessive mode of inheritance hypothesized in pedigree 2. **Table S2.** List of identified *FLNA* variants in PBS cohort. **Figure S2.** Images of PBS affected subjects hands and feet.

## Data Availability

The datasets generated during the current study are available in Clinvar under the following accession numbers: VCV000801015, VCV000800569, VCV000800568.

## Abbreviations

ACTA2: Actin, alpha 2, smooth muscle, aorta
ACTG2: Actin, gamma 2, smooth muscle, enteric
ADHD: Attention-deficit/hyperactivity disorder
chILD: Chronic interstitial lung disease
CHRM3: Cholinergic receptor muscarinic 3
CIPO: Chronic intestinal pseudoobstruction
CKD: Chronic kidney disease
CSBS: Chronic short bowel syndrome
FLNA: Filamin A
FLNB: Filamin B
FLNC: Filamin C
FMD: Frontotmetaphyseal dysplasia
GERD: Gastroesophageal reflux disease
GOF: Gain of function
HNF1b: Hepatocyte nuclear factor 1 β
Ig: Immunoglobulin repeat
ITGB1: Integrin β 1
ITGα: Integrin alpha
LOF: Loss of function
MAF: Minor allele frequency
MNS: Melnick-Needles syndrome
OPD1/2: Ototopalatodigital syndrome type 1/2
OPDSD: Otopalatodigital spectrum disorders
PBS: Prune Belly Syndrome
PVNH: Periventricular nodular heterotopia
RUBACE: Renal, ureter, bladder/outlet, abdominal wall, cryptorchidism, extra-genitourinary
S/P: Status post
STIM1: Stromal interaction molecule 1 precursor
TAA: Thoracic aortic aneurysm
VUR: Vesicoureteral reflux
WES: Whole exome sequencing
XCVD: X-linked cardiac valvular dystrophy
