# Supplementary material for: Prune belly syndrome in surviving males can be caused by Hemizygous missense mutations in the X-linked Filamin A gene
Source: BMC Med Genet. 2020 Feb 21;21:38. doi: 10.1186/s12881-020-0973-x (PMC7035669; doi:10.1186/s12881-020-0973-x)
Supplement: Supplementary file 1 — Additional file 1: Table S1. Whole exome sequencing metrics and variant calling. Figure S1. X-linked recessive mode of inheritance hypothesized in pedigree 2. Table S2. List of identified FLNA variants in PBS cohort. Figure S2. Images of PBS affected subjects hands and feet. [file 12881_2020_973_MOESM1_ESM.docx]

**Supplementary Table 1.** **Whole exome sequencing metrics and variant calling.**

| **Individual** | **Subject 1** | **Subject 2** | **Subject 3** | **Subject 4** |
| --- | --- | --- | --- | --- |
| Total Reads | 87,500,794 | 112,560,388 | 112,355,048 | 130,007,992 |
| Passed filter unique aligned reads (%) | 99.6% | 99.6% | 99.8% | 99.7% |
| Mean target coverage | 43.1 | 53.7 | 135.9 | 143.9 |
| Target coverage >20x (%) | 77.1% | 80.0% | 87.2% | 98.7% |
| Homozygous autosomal variants meeting filtering criteria ^a^ | none shared | none shared | none | none |
| Hemizygous  X-chromosome variants meeting filtering criteria ^b^ | *FLNA* (p.Cys2160Arg) | *FLNA* (p.Cys2160Arg) | *FLNA* (p.Ala1448Val)  *TIMP1* (p.Gly76Glu)  *DMD* (p.Ala305Val) | *FLNA* (p.Glu2236Asp)  *UPF3B* (p.Asp282Lys) |

^a^ Coding, ExAC AF <0.005, genotyping quality >70%

^b^ Coding, ExAC <0.00005, genotyping quality >70%

­
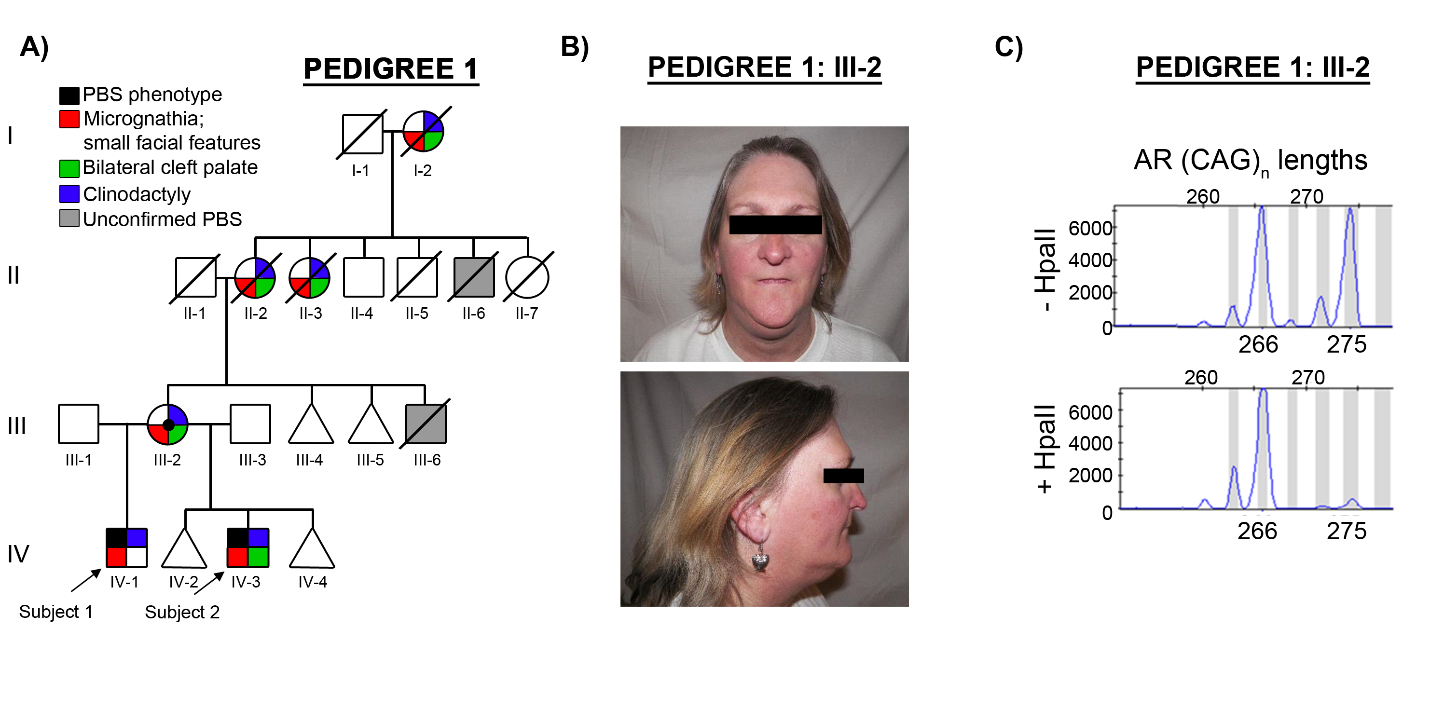


**Supplementary Figure 1. X-linked recessive mode of inheritance hypothesized in pedigree 2.**

1. Expanded pedigree 1 shows three generations of PBS on maternal side of family and multiple miscarriages. Familial history of PBS is noted in individuals II-6 (died 2 days after birth) and III-6 (died at 2 weeks) but unconfirmed. Multiple miscarriages are noted (III-4 (gender unknown), III-5 (female) and IV-4 (gender unknown)). Family history suggests that individuals I-2, II-2 and II-3 displayed OPDSD features including micrognathia, bilateral cleft palate and clinodactyly. No DNA was available from these additional family members to test for presence of the variant.
2. Photograph of III-2 from Pedigree 1, the mother of subjects 1 and 2. Note that she displays less prominent OPDSD features than her two sons including wide-set/downward slating eyes, hypodontia and micrognathia.
3. Androgen receptor (AR) methylation assay for maternal X-chromosome inactivation. 500ng DNA was digested with HpaII or mock digested and 100mg was used for PCR amplification of the AR (CAG)_n_ repeat region using 5’ 6-carboxyfluorescein labeled forward 5’-GCTGTGAAGGTTGCTGTTCCTCAT and reverse 5’-TCCAGAATCTGTTCCAGAGCGTGC primers. After PCR product separation (ABI Prism 3100 Genetic Analyzer), the total peak areas were determined for each AR allele length with or without methylation-sensitive HpaII digestion (GeneMapper V3.7), confirming maternal X-chromosome inactivation by preferential methylation of the 266bp allele in individual III-2 from pedigree 1.

**Supplementary Table 2. List of identified *FLNA* variants in PBS cohort.**

| **Individual** | **FLNA variant** | **ExAC AF** | **GERP ^a^**  **(-12.3-6.17)** | **Polyphen score (0-1)** | **Segregation analysis** |
| --- | --- | --- | --- | --- | --- |
| Subject 1 | p.C2160R | Not reported | 5.58 | 0.972 | Maternally inherited |
| Subject 2 | p.C2160R | Not reported | 5.58 | 0.972 | Maternally inherited |
| Subject 3 | p.A1448V | Not reported | 5.69 | 0.055 | Maternally inherited |
| Subject 4 | p.C2236E | Not reported | 5.44 | 1.0 | Maternally inherited |
| Subject 5 | p.R24L | Not reported | 3.42 | 0.0 | Maternally inherited and present in unaffected brother |
| Subject 6 | p.G2138C | 0.00009 | 5.64 | 1.0 | Maternally inherited and present in unaffected brother |

^a^ GERP (Genomic evolutionary rate profiling)


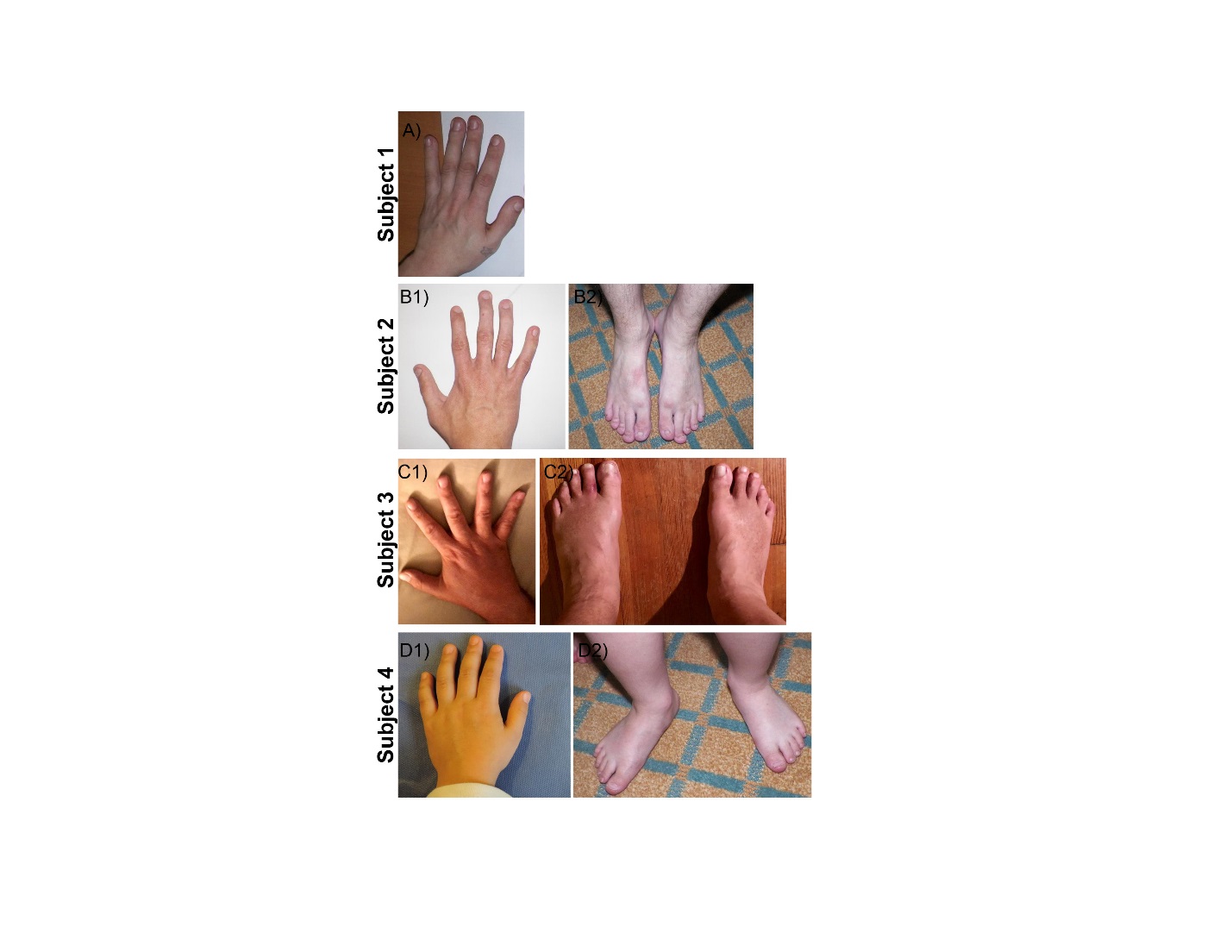


**Supplementary Figure 2. Images of PBS affected subjects hands and feet.**

A1) Subject 1 has hypoplastic distal phalanges.

B1-B2) Subject 2 has short proximally placed thumbs, hypoplastic distal phalanges, hypoplasia of the great toe, and long second toe. Note that he also exhibits “tree-frog feet” a phenotype common to OPD.

C1-C2) Subject 3 has hypoplastic distal phalanges and hypoplasia of the great toe.

D1-D2) Subject 4 exhibits hypoplastic distal phalanges and hypoplasia of the great toe.
